# Impaired spatial and contextual memory formation in *galectin-1 *deficient mice

**DOI:** 10.1186/1756-6606-4-33

**Published:** 2011-09-01

**Authors:** Masanori Sakaguchi, Maithe Arruda-Carvalho, Na Hyea Kang, Yoichi Imaizumi, Françoise Poirier, Hideyuki Okano, Paul W Frankland

**Affiliations:** 1 Program in Neurosciences and Mental Health, The Hospital for Sick Children, Toronto, Canada, M5G 1X8; 2 Department of Physiology, University of Toronto, Toronto M5S 1A8, Canada; 3 Institute of Medical Science, University of Toronto, Toronto M5S 1A8, Canada; 4 Department of Physiology, Keio University School of Medicine, Tokyo, Japan; 5 Institut Jacques Monod, Université Paris Diderot, Paris, France

## Abstract

Galectins are a 15 member family of carbohydrate-binding proteins that have been implicated in cancer, immunity, inflammation and development. While galectins are expressed in the central nervous system, little is known about their function in the adult brain. Previously we have shown that galectin-1 (gal-1) is expressed in the adult hippocampus, and, in particular, in putative neural stem cells in the subgranular zone. To evaluate how gal-1 might contribute to hippocampal memory function here we studied *galectin-1 *null mutant (gal-1^-/-^) mice. Compared to their wildtype littermate controls, gal-1^-/- ^mice exhibited impaired spatial learning in the water maze and contextual fear learning. Interestingly, tone fear conditioning was normal in gal-1^-/- ^mice suggesting that loss of *gal-1 *might especially impact hippocampal learning and memory. Furthermore, gal-1^-/- ^mice exhibited normal motor function, emotion and sensory processing in a battery of other behavioral tests, suggesting that non-mnemonic performance deficits are unlikely to account for the spatial and contextual learning deficits. Together, these data reveal a role for galectin-carbohydrate signalling in hippocampal memory function.

## Introduction

The biological actions of carbohydrate molecules are mediated, in part, by interactions with lectins which recognize carbohydrate structures and bind to their specific sequences [1,2]. Galectin-1 (gal-1) is a family member of a specific class of lectins, galectins, which regulate various biological functions through binding to lactosamine containing carbohydrate molecules [3-5]. While previous cell culture studies have identified a role for gal-1 in cell death, cell adhesion and neurite outgrowth [5,6], few studies have studied the role of this lectin in central nervous system *in vivo *[7-11].

Previously we have shown that gal-1 is expressed in neural stem cells (NSCs) and regulates neurogenesis in the adult mouse brain [7-10]. In the subventricular zone (SVZ), loss of *gal-1 *leads to reduced adult neurogenesis, suggesting that gal-1 usually promotes proliferation of the adult SVZ NSCs [7,9]. Consistent with these findings, we have found that administration of gal-1 promotes adult SVZ neurogenesis and functional recovery following ischemic brain injury [8,9]. Conversely, inhibiting gal-1 blocks ischemia-induced upregulation of SVZ neurogenesis and associated functional recovery [8]. Similarly, gal-1 potentiates the therapeutic effects of transplanted NSCs on recovery from spinal cord injury in non-human primates [12].

Gal-1 is also expressed in putative NSCs in the subgranular zone (SGZ) of the hippocampus [10,13]. However, in contrast to SVZ neurogenesis, we found that loss of *gal-1 *led to increased levels of SGZ neurogenesis in adult mice in a C57BL/6 background [10], suggesting that gal-1 may usually down-regulate neurogenesis in the adult hippocampus. As the hippocampus plays a central role in learning and memory, this raises the possibility that gal-1 contributes to behavioral plasticity either via neurogenic or non-neurogenic (as gal-1 is also expressed in mature neurons in the CA1 and CA3 regions of the hippocampus) mechanisms. To evaluate this possibility here we characterize gal-1^-/- ^mice in a range of learning and memory tasks, and we find that hippocampus-dependent contextual and spatial learning is deficient in these mice. These experiments reveal an important role for gal-1 in hippocampus-dependent learning and memory, and, more generally, represent a first step toward understanding how lectin-carbohydrate signalling contributes to hippocampal memory function.

## Results

### gal-1 is expressed in neurogenic and non-neurogenic regions in the hippocampus

**Figure 1:**
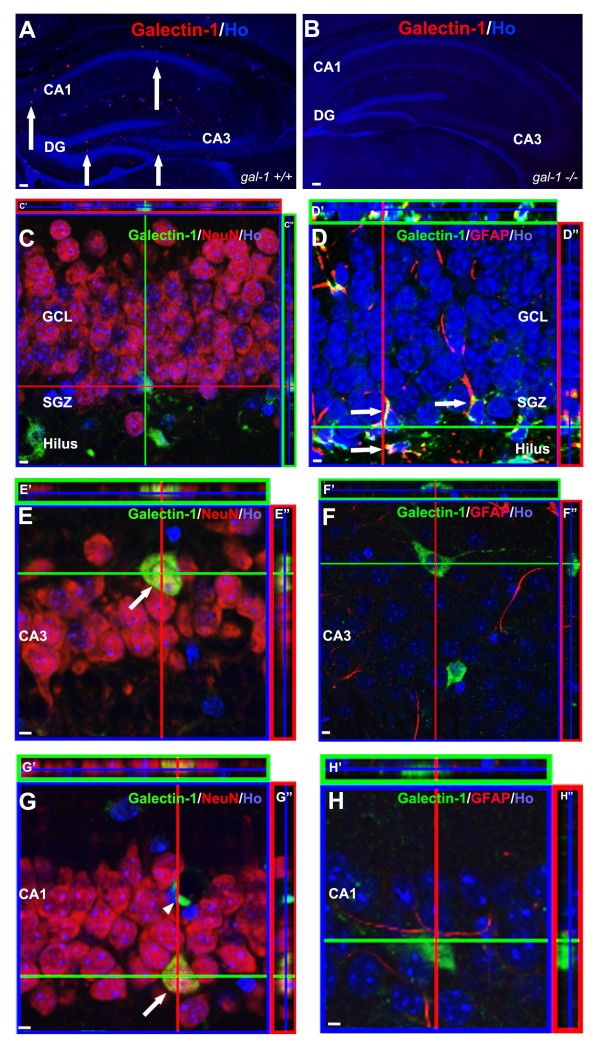
**Characterization of gal-1 expression in adult mouse hippocampus**. (A) In wild type hippocampus, gal-1 expression (red; arrows) was found in the DG, CA3 and CA1. Blue = nuclei visualized by Hoechst 33258. (B) In gal-1^-/- ^mice, the signal was completely absent indicating the specificity of our gal-1 staining. (C) In the GCL of the DG, gal-1 signal was never co-localized in NeuN-positive cells. (D) In the GCL, the vast majority of gal-1 positive cells were GFAP-positive (arrows). (E and F) In the CA3, gal-1 positive cells were always NeuN-positive (E, arrow), but not GFAP-positive (F). (G and H) In the CA1, gal-1 positive cells were either NeuN-positive (G, arrow) or NeuN-negative/GFAP-negative (G, arrowhead, H). Scale bars: (A-B), 100 μm; (C-H), 5 μm.

In order to characterize gal-1 expression in the adult hippocampus, we conducted a series of immunohistochemical analyses in adult wild-type (WT) mice. We first examined the specificity of our gal-1 antibody by staining WT vs. gal-1^-/- ^mice (Figure 1A-B). In the hippocampus (and elsewhere in the brain) we found no evidence of staining in gal-1^-/- ^mice [9,10], suggesting that this antibody binds only gal-1, and not, for example, other members of the galectin family which exhibit structural similarity in their lectin-binding domain [5,14,15]. In WT mice, gal-1 staining was detected in all three major subdivisions of the hippocampus (the dentate gyrus (DG), CA3 and CA1) (Figure 1A). In the DG, as shown before [10], we found NeuN-positive cells expressing gal-1 only in the hilus, suggesting that gal-1 is not expressed in mature neurons in the granule cell layer. In the granule cell layer, gal-1 expression was limited to GFAP-positive cells (Figure 1C-D), suggesting that gal-1 is localized to astrocytes and/or putative neural stem cells, as we have previously reported [10]. Within the CA3 region, the vast majority of cells expressing gal-1 were NeuN-positive, but never GFAP-positive (Figure 1E-F), indicating that gal-1 is expressed in mature neurons. Within the CA1 region, gal-1-expressing cells were either NeuN-positive (suggesting that they were mature neurons) or both NeuN-negative and GFAP-negative (Figure 1G-H). Based on morphology and localization pattern (flattened and semilunar cell body surrounding blood vessels (Figure 1G, arrowhead)), this latter class of cell is most likely to be endothelial cells of blood vessels as previously described elsewhere [16].

### gal-1 null mutant mice have deficient contextual fear memory

**Figure 2:**
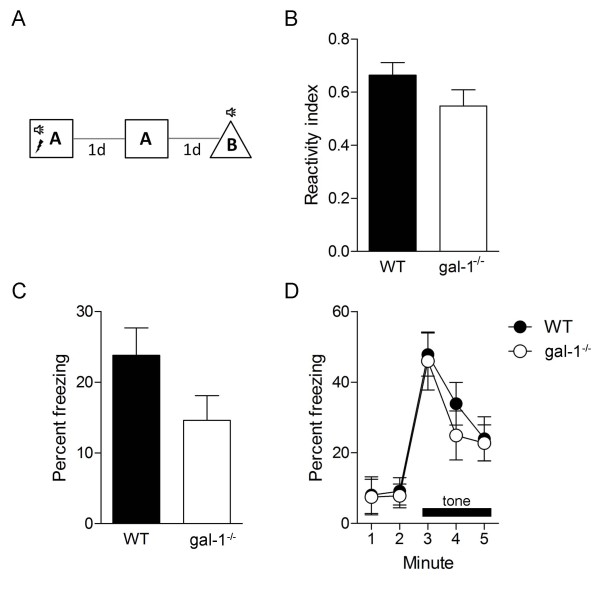
**Contextual fear memory deficits in gal-1^-/- ^mice**. (A) Experimental design used to fear condition WT (*n *= 11) and gal-1^-/- ^(*n *= 15) mice. (B) Shock reactivity in WT (black bar) vs. gal-1^-/- ^(white bar) mice. (C) Freezing in context test in WT (black bars) vs. gal-1^-/- ^(white bar) mice. (D) Freezing in tone test in context B (WT, closed circles; gal-1^-/- ^mice, open circles). The tone was presented after 120 s.

Our immunohistochemical analyses indicated that gal-1 is expressed in the adult hippocampus and, in particular, in putative NSCs in the DG [10]. Given the role of the hippocampus, and, in particular, hippocampal neurogenesis, in learning and memory [17-20], we next tested gal-1^-/- ^mice in a range of learning paradigms that depend on hippocampal function. To do this we first used a fear conditioning paradigm, in which a tone is paired with a mild foot shock in a novel context (Figure 2A). When replaced in the original training context or presented with the tone in an alternate context, mice exhibit a range of species-typical behaviors including freezing (the cessation of all but respiratory-related movement)[21]. During training, gal-1^-/- ^and WT littermate control mice showed similar reactivity to the shock (Figure 2B, unpaired t-test: *t*(19) = 1.49, *P *= 0.08), indicating that the mutant mice were able to sense the foot shock normally. Twenty-four hours later, freezing in the original training context was reduced in gal-1^-/- ^mice compared to WT littermate controls (Figure 2C, unpaired t-test: *t*(24) = 1.75, *P *< 0.05). In contrast, WT and gal-1^-/- ^mice exhibited equivalent freezing when presented with the tone in an alternate context (Figure 2D; time × genotype ANOVA, no main effect of genotype *F*(1, 21) = 0.16, *P *= 0.69, main effect of time only *F*(4, 21) = 41.59, *P *< 0.001), exhibiting similar levels of freezing before (planned comparison of WT vs. gal-1^-/- ^freezing; *t*(21) = 0.17, *P *= 0.43) and during (planned comparison of WT vs. gal-1^-/- ^freezing; *t*(21) = 0.47, *P *= 0.32) tone presentation. As the formation of contextual, but not tone, fear memories depends on hippocampal function [21], these results suggest that loss of *gal-1 *impairs the formation of hippocampus-dependent memory.

### Intensive training overcomes contextual fear memory deficits in gal-1^-/- ^mice

**Figure 3:**
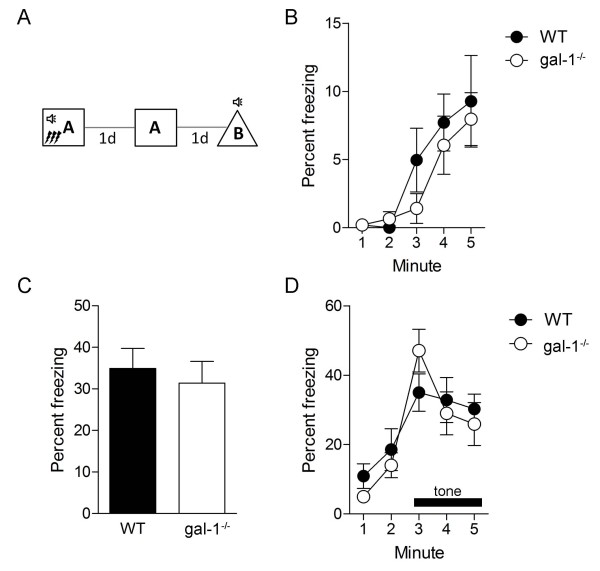
**Normal contextual fear conditioning in gal-1^-/- ^mice after strong training**. (A) Experimental design used to fear condition (3 tone-shock pairs) WT (*n *= 10) and gal-1^-/- ^(*n *= 12) mice. (B) Freezing during training in WT (closed circles) and gal-1^-/- ^mice (open circles). (C) Freezing in context test in WT (black bars) vs. gal-1^-/- ^(white bar) mice. (D) Freezing in tone test in context B (WT, closed circles; gal-1^-/- ^mice, open circles). The tone was presented after 120 s.

We next asked whether more intensive training might overcome these deficits in contextual fear memory in gal-1^-/- ^mice. To do this, during training mice received 3 tone-shock pairings (Figure 3A). During training, freezing increased incrementally, and levels were similar in both WT and gal-1^-/- ^mice (Figure 3B, time × genotype ANOVA, no main effect of genotype *F*(1, 20) = 0.65, *P *= 0.43, main effect of time only *F*(4, 20) = 12.34, *P *< 0.001). When placed back in the training context test one day later, WT and gal-1^-/- ^mice froze at similar levels (Figure 3C, unpaired t-test: *t*(20) = 0.49, *P *= 0.32) indicating that more intensive training can overcome contextual fear deficits in gal-1^-/- ^mice. Furthermore, freezing in the tone test was similar in both groups (Figure 3D, time × genotype ANOVA, main effect of time only *F*(4, 20) = 19.72, *P *< 0.001), with similar levels of freezing before (planned comparison; *t*(20) = 0.19, *P *= 0.43) and during (planned comparison; *t*(21) = 1.14, *P *= 0.13) tone presentation in WT and gal-1^-/- ^mice. These data indicate that more intensive training may overcome these deficits in gal-1^-/- ^mice perhaps because other galectins may compensate for the loss of *gal-1 *or other brain regions may be recruited to support learning when multiple shocks are used [22,23].

### gal-1^-/- ^mice have deficient spatial learning in the water maze

**Figure 4:**
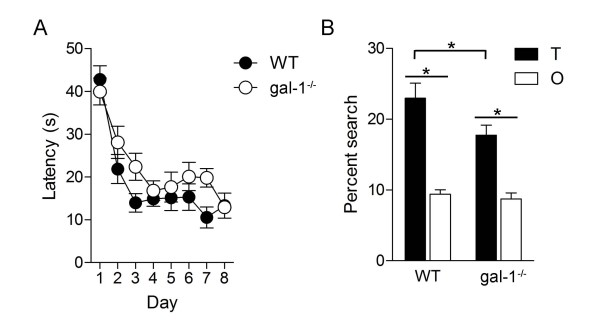
**Spatial learning deficits in gal-1^-/- ^mice in the hidden platform version of the water maze**. (A) Escape latencies during training for WT (*n *= 14; closed circles) and gal-1^-/- ^(*n *= 15; open circles) mice. (B) Probe test data showing percent time searching target (T) zone (black bars) vs. average of three other equivalent zones (white bars) for WT and gal-1^-/- ^mice (* < 0.05).

The contextual fear conditioning deficits are consistent with the idea that loss of *gal-1 *impacts hippocampal learning and memory. To evaluate whether these deficits generalize to another form of hippocampus-dependent learning, we next trained mice in the hidden version of the water maze [24,25]. During training, while latencies to locate the hidden platform decreased in both groups of mice, there was a strong tendency for longer latencies in gal-1^-/- ^mice (Figure 4A, time × genotype ANOVA, main effect of time *F*(7, 27) = 22.89, *P *< 0.001, main effect of genotype *F*(1, 27) = 4.05, *P *= 0.054). As the adoption of either localized/spatially-precise (e.g., focal searching) or some non-localized/spatially-imprecise (e.g., chaining) search strategies may contribute to reduced escape latencies across training [26-28], latency data can be poor predictors of spatial memory [26]. To better evaluate spatial memory, mice were given a probe test with the escape platform removed from the pool. In this test, both WT and gal-1^-/- ^mice searched selectively, spending more time in the region of the pool that formerly contained the platform (target [T] zone) compared to other equivalent regions of the pool (average of all other [O] zones) (Figure 4B, T > O for WT [planned comparison: *t*(13) = 5.34, *P *< 0.001] and gal-1^-/- ^[planned comparison: *t*(14) = 7.34, *P *< 0.001] mice). However, the degree of selectivity was greater in WT compared to mutants (Figure 4B, T_WT _> T_gal-1-/-_; unpaired t-test: *t*(27) = 2.06, *P *< 0.05), indicating that while gal-1^-/- ^mice were able to form a spatial memory it was nonetheless not as precise as in WT littermate controls. Consistent with the fear conditioning analysis, these results suggest that loss of *gal-1 *impairs hippocampal memory formation.

### General behavior is not altered in gal-1^-/- ^mice

**Figure 5:**
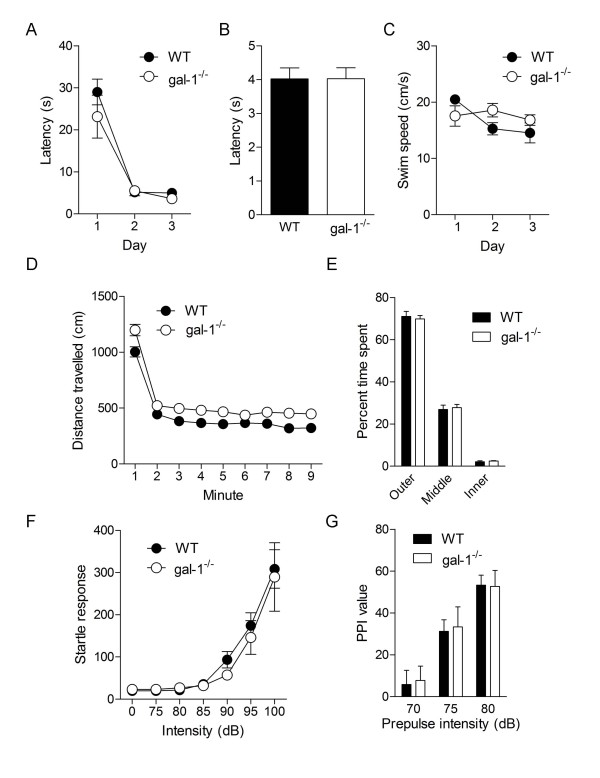
**General behavior in gal-1^-/- ^mice**. (A) Latencies to find platform for WT (*n *= 3; closed circles) vs. gal-1^-/- ^mice (*n *= 3; open circles) in the visible version of the water maze. (B) Latency to locate visible platform moved to opposite quadrant for WT (*n *= 3; black bar) and gal-1^-/- ^(*n *= 3; white bar) mice. (C). Swim speed for WT (*n *= 3; closed circles) vs. gal-1^-/- ^mice (*n *= 3; open circles) across training days in the visible version of the water maze. (D) Distance travelled in open field for WT (*n *= 12; black circles) vs. gal-1^-/- ^mice (*n *= 17; white circles). (E) Exploratory activity in outer, middle and inner regions of an open field for WT (black bars) vs. gal-1^-/- ^mice (white bars) in open field test. (F) Startle responses evoked by different intensity noise bursts (0, 75-100 dB) for WT (*n *= 13; closed circles) vs. gal-1^-/- ^mice (*n *= 8; open circles). (G) Prepulse inhibition using three different intensity prepulses WT (*n *= 13; black bars) vs. gal-1^-/- ^mice (*n *= 8; white bars).

Because gal-1 is also expressed outside of the hippocampus [11,29] we next evaluated whether other types of behaviors are altered in gal-1^-/- ^mice. First, mice were trained in a visual version of the water maze, where the platform location is marked by a cue. During training, WT and gal-1^-/- ^mice were equivalently efficient in finding the platform (Figure 5A, time × genotype ANOVA, no main effect of genotype *F*(1, 4) = 2.10, *P *= 0.22, main effect of time only *F*(2, 8) = 39.63, *P *< 0.001). At the end of training, the platform and cue were moved to the opposite quadrant, and mice were retested. As before, latencies to reach the platform were equivalent in WT and gal-1^-/- ^mice (Figure 5B; unpaired t-test: *t*(4) = 0.01, *P *= 0.50), suggesting that the ability to associate the cue with the platform location is not compromised in gal-1^-/- ^mice. Furthermore, equivalent performance in this visual version of the water maze suggests that general motor function, vision and motivation are not altered in gal-1^-/- ^mice. Consistent with this, swim speeds across trials were similar in both groups (Figure 5C, time × genotype ANOVA, no main effect of genotype *F*(1, 4) = 0.43, *P *= 0.55). Second, in an open field test, while gal-1^-/- ^mice were generally more active (Figure 5D, time × genotype ANOVA, main effects of genotype *F*(1, 27) = 10.35, *P *< 0.01 and time *F*(8, 27) = 271.5, *P *< 0.001), the spatial distribution of activity was similar in WT and gal-1^-/- ^mice suggesting that loss of *gal-1 *does not alter general anxiety levels (Figure 5E, zone × genotype ANOVA, no main effect of genotype *F*(1, 27) = 2.10, *P *= 0.16, main effect of zone only *F*(2, 27) = 660, *P *< 0.001). Third, sensory-motor function, assessed using a startle reflex paradigm [30], appears to be normal in gal-1^-/- ^mice. Startle threshold (Figure 5F, 85 dB > 0 dB for WT [*P *< 0.05], 90 dB > 0 dB for gal-1^-/- ^[*P *< 0.05]) and magnitude (Figure 5F, intensity × genotype ANOVA, no main effect of genotype *F*(1, 19) = 0.23, *P *= 0.64, main effect of intensity only: *F*(6, 114) = 36.88, *P *< 0.001) were similar in WT and gal-1^-/- ^mice. Moreover, inhibition of the startle response by auditory prepulses was unaltered in gal-1^-/- ^mice (Figure 5G; intensity × genotype ANOVA, no main effect of genotype *F*(1, 19) = 0.02, *P *= 0.90, main effect of intensity only: *F*(2, 38) = 94.94, *P *< 0.001), suggesting that the processing of extraneous auditory stimuli is normal following loss of *gal-1*. Taken together, these results suggest that impaired spatial and contextual memory formation in gal-1^-/- ^mice cannot be attributed to non-specific impact of this mutation on general behavior, emotion or sensory processing.

## Discussion

While galectins are expressed in the adult nervous system, little is known about how they contribute to adult brain function. Using a *gal-1 *null mutant mouse model, here we explored the role of gal-1 in hippocampal learning and memory. We found that loss of *gal-1 *was associated with impaired learning in two hippocampus-dependent tasks, contextual fear conditioning and spatial learning in the water maze. Additional control experiments revealed no obvious changes in motor function, emotion and sensory processing, suggesting that non-mnemonic performance factors cannot account for the learning deficits in gal-1^-/- ^mice. Together, these studies reveal a role for gal-1, and, more broadly, lectin-carbohydrate signalling in hippocampus-dependent learning and memory.

Our behavioral analyses revealed that loss of *gal-1 *impaired two forms of hippocampal learning. However, the mechanisms underlying these effects are unclear and there are several possibilities that may be evaluated in future studies. Previously we showed that gal-1 regulates adult neurogenesis by regulating the proliferation of neural stem cells [9]. As adult neurogenesis in the hippocampus very likely contributes to learning and memory [17,18], one possibility is that altered regulation of adult neurogenesis is responsible for the observed learning deficits in gal-1^-/- ^mice. In gal-1^-/- ^mice, adult neurogenesis is increased in the hippocampus (but decreased in the SVZ). However, more typically a reduction (rather than an increase) in adult neurogenesis has been associated with impaired hippocampal memory (e.g., [17,31-34], but see:[35]). This suggests that if there is a causal relationship between gal-1-related decreases in neurogenesis and the observed contextual and spatial learning deficits it is unlikely to be straightforward. Nonetheless, in gal-1^-/- ^mice it is possible that while additional cells are generated, they do not integrate normally into hippocampal networks and such aberrant integration might lead to disruption of hippocampal memory function [36,37]. To explore whether such a dominant-negative effect is plausible it will be necessary to characterize the morphological maturation of these neurons as well as their potential to become functionally integrated into hippocampal memory circuits [38-40].

An alternate possibility is that loss of *gal-1 *affects synaptic plasticity in the hippocampus. For example, in adult NSCs gal-1 promotes proliferation by binding β1 integrin [7]. As (i) gal-1 is also expressed in mature neurons in both CA3 and CA1 and (ii) β1 integrin signalling plays key roles in synaptic plasticity (e.g., LTP [41]) and hippocampus dependent memory [42] possibly through AMPA receptor trafficking and glycine receptor lateral diffusion [43], it is possible that loss of *gal-1 *impacts synaptic plasticity mechanisms in mature hippocampal neurons. A third possibility is that hippocampal wiring is altered in gal-1^-/- ^mice. For example, we have previously shown that gal-1 promotes neurite extension in neurons derived from human NSCs [12]. Since gal-1 can be externalized from the cell membrane, it is possible that the gal-1 expression in the developing hippocampus affects circuit formation, which could compromise memory stability.

## Conclusions

Our knowledge of how carbohydrate molecules exert their biological actions is currently very limited [1,2]. This is in part due to the difficulty of identifying and manipulating carbohydrate molecules *in vivo*. However, such limitations may be overcome by genetically targeting upstream enzymes responsible for production of carbohydrate structures or downstream binding partners such as lectins which recognize and bind to specific structural portion of carbohydrates [44,45]. These approaches have revealed critical roles of carbohydrate molecules in many different biological contexts in animals from fertilization to the patterning of neuronal wiring [44,45]. Adopting the latter approach, our behavioral characterization of the gal-1^-/- ^mice represents a first step towards understanding how lectin-carbohydrate signalling might contribute to hippocampal learning and memory. They reveal a specific role for gal-1 in learning and memory, and, as binding of gal-1 to several lactosamine derivatives has been well-characterized [14], future studies may focus on mechanisms underlying these effects.

## Methods

### Mice

We used gal-1^-/- ^mice that had been backcrossed > 10 generations onto the C57BL6/J background, as described previously (Imaizumi et al., 2011). For the study, 8 to 13-week old mice were used throughout the study and killed by anesthetic overdose at the end of each experiment. We used wild type littermates mice as controls, and similar numbers of male and female mice were used in each experiment. Mice were maintained on a 12-h/12-h light/dark cycle with unlimited access to food and water. All the experiments were performed in accordance with guidelines and regulations of The Hospital for Sick Children, Animal Care and Use Committee.

### Immunohistochemistry

Brains were perfusion-fixed with 4% paraformaldehyde (PFA), postfixed in the same fixative overnight, and then cut into 50-μm sections on a vibratome (Leica). After three rinses in PBS, the sections were incubated with primary antibodies overnight, and then incubated for 60 min at room temperature with the Fab2-portion of secondary antibodies (1:500; Jackson ImmunoResearch) conjugated with HRP or biotin (Jackson ImmunoResearch). The biotin or HRP-conjugated antibodies were visualized using TSA (Pharmingen) with or without the Vectastain Elite ABC kit (Vector Laboratories). For multi-color labeling, the potential for the cross-reactivity of the secondary antibodies with off-target primary antibodies was carefully tested and excluded by using the appropriate controls (e.g., parallel staining without one of the primary antibodies). The primary antibodies (final dilution and source) used in this study were as follows: goat anti-Galectin-1 (1:200, R&D Systems), mouse monoclonal anti-GFAP (1:200, Sigma); mouse monoclonal anti-NeuN (1:100, Chemicon). All representative images were acquired using epifluorescent (Nikon Eclipse 80i) or confocal (LSM 510 Zeiss) microscopes. To calculate the proportion of double-labeled cells, confocal 1 μm Z-stack images were obtained using ZEN software (Zeiss, Germany) with a minimal interval of 15 μm to prevent duplicate counts of the same cell.

### Fear conditioning

The apparatus and behavioral procedures have been previously described [46]. In the fear conditioning experiments, two contexts were used. Context A consisted of a conditioning chamber (31 cm × 24 cm × 21 cm; Med Associates, St. Albans, VT), containing a stainless steel shock-grid floor. Shock grid bars (diameter 3.2 mm) were spaced 7.9 mm apart. The grid floor was positioned over a stainless-steel drop-pan, which was lightly cleaned with 70% ethyl alcohol to provide a background odor. The front, top, and back of the chamber were made of clear acrylic and the two sides made of modular aluminum. For context B, a white, plastic floor covered the shock grid bars and a plastic, triangular insert was placed inside the same conditioning chamber used for context A. One of the walls of this insert had a black/white striped pattern. The other two walls were white. Context B was cleaned with water. As contexts A and B were located in the same windowless room and used common apparatus, they shared some overlapping features. Mouse freezing behavior was monitored via overhead cameras. Freezing was assessed using an automated scoring system (Actimetrics, Wilmette, IL), which digitized the video signal at 4 Hz and compared movement frame by frame to determine the amount of freezing.

During training, mice were placed in context A for 3 min. After 2 min of free exploration mice were presented with a 30 s tone (2800 Hz, 85 dB) that co-terminated with a 2 s footshock (0.5 mA). Mice remained in the context for a further 30 s before being returned to their home cage. Responsivity to the shock during training was estimated by comparing mouse velocity immediately preceding vs. during shock presentation using the following formula: (velocity_shock _- velocity_pre-shock_)/(velocity_shock _+ velocity_pre-shock_). Twenty-four hours after training, freezing was assessed in a 3 min test in context A. Twenty-four hours later, mice were placed in an altered context (context B). After 2 min, the tone was presented. In the intensive training protocol, mice received three tone-footshock pairings. As previously, each tone (30 s, 2800 Hz, 85 dB) co-terminated with a 2 s footshock (0.5 mA). Tones were presented after 120 s, 180 s and 240 s. Mice were returned to the home cage 30 sec after the final foot shock.

### Hidden version of the water maze

The apparatus and behavioral procedures have been previously described [25]. Behavioral testing was conducted in a circular water maze tank (120 cm in diameter, 50 cm deep), located in a dimly-lit room. The pool was filled to a depth of 40 cm with water made opaque by adding white, non-toxic paint. Water temperature was maintained at 28 ± 1°C by a heating pad located beneath the pool. A circular escape platform (10 cm diameter) was submerged 0.5 cm below the water surface, in a fixed position in one of the quadrants. The pool was surrounded by curtains, at least 1 m from the perimeter of the pool. The curtains were white and had distinct cues painted on them.

Water maze training took place over 8 days. On each day mice received 3 training trials (inter-trial interval was ~15 s). On each trial, mice were placed into the pool, facing the wall, in one of 4 pseudorandomly-varied start locations. The trial was complete once the mouse found the platform or 60 seconds had elapsed. If the mouse failed to find the platform on a given trial, the experimenter guided the mouse onto the platform. Twenty-four hours following the completion of training, spatial memory was assessed in a 60 s probe test with the platform removed from the pool. Behavioral data from training and the probe tests were acquired and analyzed using an automated tracking system (Actimetrics, Wilmette, IL). Using this software, we recorded parameters during training, including escape latency and swim speed. In probe tests, we measured the amount of time mice searched the target zone (23.6 cm in radius, centered on the location of the platform during training) vs. the average of three other equivalent zones in other areas of the pool. Each zone represents 15% of the total pool surface.

### Visual version of the water maze

To control for sensory and motor impairments, we trained mice in the visual version of the water maze. In this version, platform location was marked by a cylindrical cue (4 cm in diameter, 4 cm in height), with a vertical black/white striped pattern. Mice were trained for 3 days (6 trials/day). On each trial, the platform location was kept constant across trials while the start position was varied pseudo-randomly. Forty-eight hours after the completion of training, the platform and cue were re-positioned in the opposite quadrant of the pool, and the mice were given a single trial. The latency to reach the platform and swim speed were recorded.

### Open Field

Mice were placed in the center of a square-shaped open field (45 cm × 45 cm × 20 cm height) and allowed to explore for 10 min. The open field apparatus was constructed of Plexiglas, and was dimly-lit from above. Mouse location was tracked by a camera located above. Total distance travelled and time spent in 3 different zones (outer, middle, inner) were measured (Limelight2, Actimetrics, Wilmette, IL). Distribution of activity in different regions of the arena was used as a measure of anxiety-related behavior [47].

### Acoustic startle experiments

Startle testing was conducted in a MEDASR-310 startle testing system (MedAssociates, VT, USA). Mice were placed in a Plexiglas cylinder (3.2 cm internal diameter) for testing. Acoustic startle stimuli and prepulse stimuli were delivered via a high-frequency speaker, placed at a distance of 15 cm from the testing cylinder. Background noise levels were maintained at 65 dB throughout the experiments. The testing cylinder was mounted on a sensor platform. A piezoelectric accelerometer, attached to the base of the sensor platform, detected and transduced all cage movements, and these were recorded by a computer. The startle amplitude was taken to be the maximal response occurring within 100 ms of the presentation of the startle stimulus. The speakers, testing cylinder and sensor platform were housed within a sound-attenuated chamber.

Mice were initially given a habituation session to acclimate them to the testing environment. In this session, mice were presented with 80 startle stimuli, delivered at a fixed intertrial interval of 15 s. The startle stimulus was a 40 ms, 120 dB noise burst with a rise/fall time of less than 1 ms. One day following this, prepulse inhibition was tested. Following an acclimation period of 5 min, mice were initially presented with a total of 20 noise bursts (40 ms duration, 120 dB, 1 ms rise/fall time). In the prepulse inhibition phase, mice were presented with a total of 90 trials. Three prepulse intensities were tested: 70, 75 and 80 dB. Prepulses were 20 ms in duration with a rise/fall time of less than 1 ms. For each prepulse intensity, there were three types of trial: prepulse alone, prepulse/startle stimulus and startle stimulus alone. In the prepulse/startle stimulus trial, the onset of the prepulse preceded the onset of the startle stimulus by 100 ms. The trials were spaced 15 s apart. Percent PPI was calculated for each mouse according to the following formula:: %PPI = [1-(*Response_prepulse + startle stimulus_/Response_startle stimulus alone_*)] × 100)

Twenty four hours later, mice were given a startle threshold test session. Following an acclimation period of 5 min, mice were presented with a total of 70 trials at a fixed intertrial interval of 15 s. There were 7 trial types: no stimulus, and 6 types of trials where startle stimuli at a range of intensities were presented (75-100 dB; 5 dB increments). The startle stimuli were 40 ms noise bursts with a rise/fall time of less than 1 ms. The 7 trial types were presented in a pseudorandom order such that each trial type was presented once within a block of 7 trials. Startle threshold was defined as the minimal intensity at which responding was significantly greater than in the no stimulus (0 dB) trials.

### Statistical analysis

Values are expressed as the mean ± standard error of the mean (s.e.m.). An unpaired t-test (for two groups) or ANOVA were used to detect group differences. Because both male and female mice were used in these studies we initially included gender as a factor in our analyses. However, we found no effects of gender, and no significant interactions between gender and genotype, and therefore this factor was subsequently dropped from analysis.

## Competing interests

The authors declare that they have no competing interests.

## Authors' contributions

MS and PWF designed the research. MS, MAC, NHK and YI performed the research. HO and FP generated the gal-1^-/- ^mice. MS, MAC and PWF prepared the manuscript. All authors read and approved the final manuscript.
